# Lamina dura thickening in patients on antiresorptive therapy as a radiographic risk indicator of medication-related osteonecrosis of the jaw: a retrospective longitudinal cohort study

**DOI:** 10.1093/dmfr/twag029

**Published:** 2026-05-19

**Authors:** Sonya Radi, Samy El Bachaoui, Ilya Tsiklin, Constanza Pastenes, Hanne De Backer, Ruxandra Coropciuc, Tim Van den Wyngaert, Benoit Beuselinck, Reinhilde Jacobs

**Affiliations:** OMFS-IMPATH Research Group, Department of Imaging and Pathology, Faculty of Medicine, KU Leuven, 3000 Leuven, Belgium; OMFS-IMPATH Research Group, Department of Imaging and Pathology, Faculty of Medicine, KU Leuven, 3000 Leuven, Belgium; Department of Oral and Maxillofacial Surgery, University Hospital of Leuven, 3000 Leuven, Belgium; OMFS-IMPATH Research Group, Department of Imaging and Pathology, Faculty of Medicine, KU Leuven, 3000 Leuven, Belgium; Department of Oral and Maxillofacial Surgery, University Hospital of Leuven, 3000 Leuven, Belgium; OMFS-IMPATH Research Group, Department of Imaging and Pathology, Faculty of Medicine, KU Leuven, 3000 Leuven, Belgium; Oral and Maxillofacial Radiology Department, Faculty of Dentistry, Universidad de Los Andes, 7620157 Santiago, Chile; OMFS-IMPATH Research Group, Department of Imaging and Pathology, Faculty of Medicine, KU Leuven, 3000 Leuven, Belgium; Department of Oral and Maxillofacial Surgery, University Hospital of Leuven, 3000 Leuven, Belgium; Department of Nuclear Medicine, University Hospital of Antwerp, 2610 Wilrijk, Antwerp, Belgium; Laboratory of Experimental Oncology, KU Leuven, 3000 Leuven, Belgium; University Hospitals Leuven, Medical Oncology, 3000 Leuven, Belgium; OMFS-IMPATH Research Group, Department of Imaging and Pathology, Faculty of Medicine, KU Leuven, 3000 Leuven, Belgium; Department of Dental Medicine, Karolinska Institutet, 141 52 Huddinge, Stockholm, Sweden

**Keywords:** lamina dura, MRONJ, panoramic radiography, antiresorptive drugs

## Abstract

**Aims:**

This study investigated whether antiresorptive drug (ARD) therapy is associated with lamina dura (LD) thickening, defined as a radiographic increase in the thickness of the normally thin, radiopaque cortical line surrounding the tooth root. It further evaluated whether LD thickening serves as a radiographic marker and risk indicator for medication-related osteonecrosis of the jaw (MRONJ).

**Methods:**

Panoramic radiographs from 65 ARD-treated patients (University Hospital Leuven) and 65 age- and sex-matched controls were analyzed. Inclusion criteria were prior oncological diagnosis, high-dose intravenous ARD, and radiographs before and after therapy. Three blinded oral and maxillofacial radiologists categorized LD appearance into 4 grades: (0) not/barely visible, (1) normal, (2) mild thickening, and (3) marked thickening. Group differences and longitudinal changes were evaluated using appropriate non-parametric and time-to-event analyses.

**Results:**

Among 130 participants, LD thickness remained stable in controls but increased in ARD-treated patients (*P* < .001). Within the ARD group, LD thickening changed over time (*P* < .001): 30.6% showed no change, 55.4% developed mild thickening, and 13.8% marked thickening. Controls showed minimal change (*P* = .011), with 9.2% developing mild thickening. Kaplan-Meier analysis estimated a median time to LD thickening of 27 months, with a higher thickening probability in ARD-treated patients than controls (*P* < .001). Lamina dura thickening was not directly associated with MRONJ (*P* = .39).

**Conclusion:**

Lamina dura thickening is strongly associated with long-term ARD exposure and shows progressive changes. Although not directly associated with MRONJ in this cohort, it may still represent a potential risk indicator, warranting confirmation in further studies.

## Introduction

Antiresorptive drugs (ARDs), such as bisphosphonates and denosumab, are widely prescribed in the treatment of skeletal disorders including osteoporosis, Paget’s disease, bone metastatic cancer, and multiple myeloma.^1^^,^^2^ These agents act by inhibiting osteoclast-mediated bone resorption, reducing bone turnover, and maintaining skeletal strength.^3^^,^^4^ Bisphosphonates and denosumab are the most extensively used, among ARDs, including in oncology to prevent skeletal-related events.^3^^,^^5–7^ However, ARD treatment increases the risk of medication-related osteonecrosis of the jaw (MRONJ), a severe adverse effect marked by progressive bone destruction that could result in infection, chronic pain, and fractures.^8–10^ Medication-related osteonecrosis of the jaw was first described by Marx in 2003 in patients treated with intravenous bisphosphonates.^4^^,^^11^ The American Association of Oral and Maxillofacial Surgeons (AAOMS) later expanded the definition to include cases caused by other antiresorptive or antiangiogenic agents. The condition typically follows dental interventions or arises in inflamed areas, and its risk increases with the duration of treatments. In patients receiving low-dose ARDs for osteoporosis, the incidence of MRONJ can reach up to 0.2% when treatment duration exceeds 4 years.^12^ In contrast, high-dose ARD use in oncological settings has an incidence of 6.7% in bisphosphonate users, 10% in denosumab users, which can even reach up to 15% in patients receiving sequential treatment with both types of ARDs.^13^^,^^14^

The jawbone is particularly prone to MRONJ because of its constant exposure to microtrauma, microbes, and moist environment. The pathogenesis is not fully understood and is likely multifactorial, including inhibited bone tissue healing, compromised vascularization, mechanical stress, genetic predisposition, and bacterial colonization.^3^^,^^4^^,^^6^^,^^15^ According to AAOMS guidelines, MRONJ is diagnosed based on exposed bone or bone that can be probed through an intraoral or extraoral fistula(e) in the maxillofacial region persisting for over 8 weeks in patients without prior radiation or jaw metastases.^2^^,^^6^ These guidelines describe 4 stages, with stage 0 cases defined as prodromal, showing no clinical evidence of necrotic bone, but exhibiting nonspecific symptoms or radiographic signs, such as odontalgia without odontogenic cause, osteosclerosis, and thickened lamina dura (LD).^2^^,^^16^ Stage 1 is characterized by delayed wound healing or exposed bone; stage 2 adds signs of infection and inflammation; and stage 3 is characterized by more severe complications, such as extensive osteonecrosis, pathological fractures, and fistula formation.^9^^,^^10^

Early diagnosis is critical, as delayed detection worsens prognosis. Yet, most current clinical definitions focus on bone exposure, often identifying MRONJ at a more advanced stage.^8^ Biopsy is avoided due to impaired healing, making radiographic diagnosis represent an important tool for early assessment.^4^^,^^17^ Panoramic imaging is widely used in clinical practice and is more accessible for dentists than cone-beam CT (CBCT), which is more expensive and involves higher radiation exposure.^18^^,^^19^ While periapical images offer higher spatial resolution and might seem more suitable for assessing LD thickening, multiple images would be needed to cover the full maxilla and mandible making panoramic radiographs the more reliable option for this assessment.^18^^,^^19^ Although early imaging changes like LD thickening and osteosclerosis have been reported, their diagnostic value remains uncertain due to the lack of consensus and validated radiographic criteria.^1^^,^^4^^,^^8^^,^^20^ The incidence of LD thickening in MRONJ patients varies between studies but is in general significantly higher in patients using ARDs. Kubo et al. showed in 2018 that LD thickening was observed more frequently in Japanese patients using bisphosphonates compared to unmedicated patients.^20^ Moreno-Rabié et al.^2^ showed a significant difference in incidence of LD thickening between patients treated with ARDs (10%) compared to the control group (3%). As LD changes can be caused by several factors (eg, trauma, orthodontic movement, periodontal disease), these studies lack confirmation that LD thickening is merely caused by ARD use and that it was not already present due to other reasons.^21^^,^^22^ Furthermore, past studies regarding this topic did not include radiographs prior to ARD use.^23–28^

The primary objective of this study was to evaluate the impact of ARDs (including both bisphosphonates and denosumab) on the thickness of the LD, which is normally a thin, radiopaque cortical line surrounding the tooth root. A subobjective was to investigate the association between LD thickening and MRONJ incidence. Identifying radiographic risk features supports earlier diagnosis and prevention, ultimately improving clinical outcomes, and deepening our understanding of MRONJ pathogenesis.

## Methods

### Study design

A retrospective longitudinal cohort study was performed. Radiological data were collected from patients treated at Gasthuisberg University Hospital in Leuven, Belgium. The study was conducted in accordance with GDPR and the Declaration of Helsinki. Ethical approval was obtained by the ethical committee of UZ Leuven (approval number: S63934). A priori power analysis was performed using R studio (Posit, Version 2025.05.0 + 496) to ascertain the minimum appropriate sample size. This analysis was predicated on an anticipated effect size of 0.4, a significance level of α = 0.05, and a desired statistical power of 1 – β  =  0.90. This analysis indicated that a minimum sample size of 40 was necessary for the study.

### Participant selection

Clinical records of 680 patients treated with ARDs, who visited the hospital as part of the dental recall program, and who underwent panoramic radiography between 2010 and 2024 were reviewed. Inclusion criteria were: (1) prior oncological diagnosis (eg, multiple myeloma, prostate, breast, or lung cancer); (2) exposure to high-dose intravenous ARDs (bisphosphonates, denosumab); and (3) availability of panoramic radiographs before (T0) and after/during ARD treatment (TX, minimum of 2 months of ARD treatment). Exclusion criteria were lack of baseline or follow-up radiographs, ARD exposure in context of bone-related disorders (eg, osteoporosis) due to lower doses, radiotherapy in the maxillofacial region, systemic bone remodeling diseases, oral carcinomas, edentulous status, or incomplete records. Only radiographs with adequate resolution and full jaw visualization were included. A control group of age- and sex-matched healthy individuals was also included. Inclusion criteria were: (1) no history of polypharmacy, antiresorptive, or antiangiogenic drug exposure; (2) no history of oncological diagnosis or other systematic diseases; and (3) availability of multiple panoramic radiographs over time. Patients were excluded if the reason of consultation could potentially affect the LD (eg, jaw fractures, orthognathic treatment, and periodontal diseases).

### Data selection

Medical records were revised, including clinical data and panoramic radiographs. The following information was retrieved: age, sex, nicotine usage, alcohol usage, primary disease, ARD type, MRONJ stage, dental hygiene, tooth extractions, polypharmacy, and ARD timeline, and radiographic description of LD changes.

### Radiological assessment

Radiographs were obtained using VistaPano S or Ceph (Dürr Dental SE) at 72 kVp, 12 mA, and 7 seconds exposure time. Images were anonymized, exported in TIFF format, and assigned random participant numbers. Prior to the evaluation, a calibration session was performed with the radiologists to avoid differences in judgment. A subset of 10 LD thickening cases and 10 controls was reviewed to ensure consistency in scoring. During this session, the observers discussed the predefined grading criteria, reviewed borderline examples, and aligned their interpretation of LD thickening. Any discrepancies were discussed until a shared understanding of the scoring protocol was reached. Three blinded oral and maxillofacial radiologists, with more than 5 years of experience in the field of diagnosing and treating MRONJ, evaluated the LD in a dimmed room with a 60 cm viewing distance using a high-resolution monitor (MD Barco MDRC-2221; Barco). The radiographic evaluation was conducted independently first and resolved by consensus, if needed. First, the appearance of the LD was classified into 4 categories (Figure 1): (0) not visible/barely visible, (1) normal appearance, (2) mild thickening, and (3) markedly thickened. A normal LD is typically observed as having a width comparable to the periodontal ligament (PDL) space, which generally measures between 100 and 200 µm.^29^ If the LD appeared approximately the same width as the PDL space, it was classified as category 1. If the LD appeared thickened, it was placed in category 2, and if clear or pronounced thickening was present, it was assigned to category 3 (Figure 3). The observations were made at the tooth level, and an overall LD score was subsequently assigned at the patient level. Secondly, the radiologists were asked to describe categorical LD changes going from the T0 to the TX radiograph categorizing them as “no change” (0/1/2/3 -> 0/1/2/3), “thickening” (0->1,1->2, 2->3), or “severe thickening” (0->2/3, 1->3).

**Figure 1:**
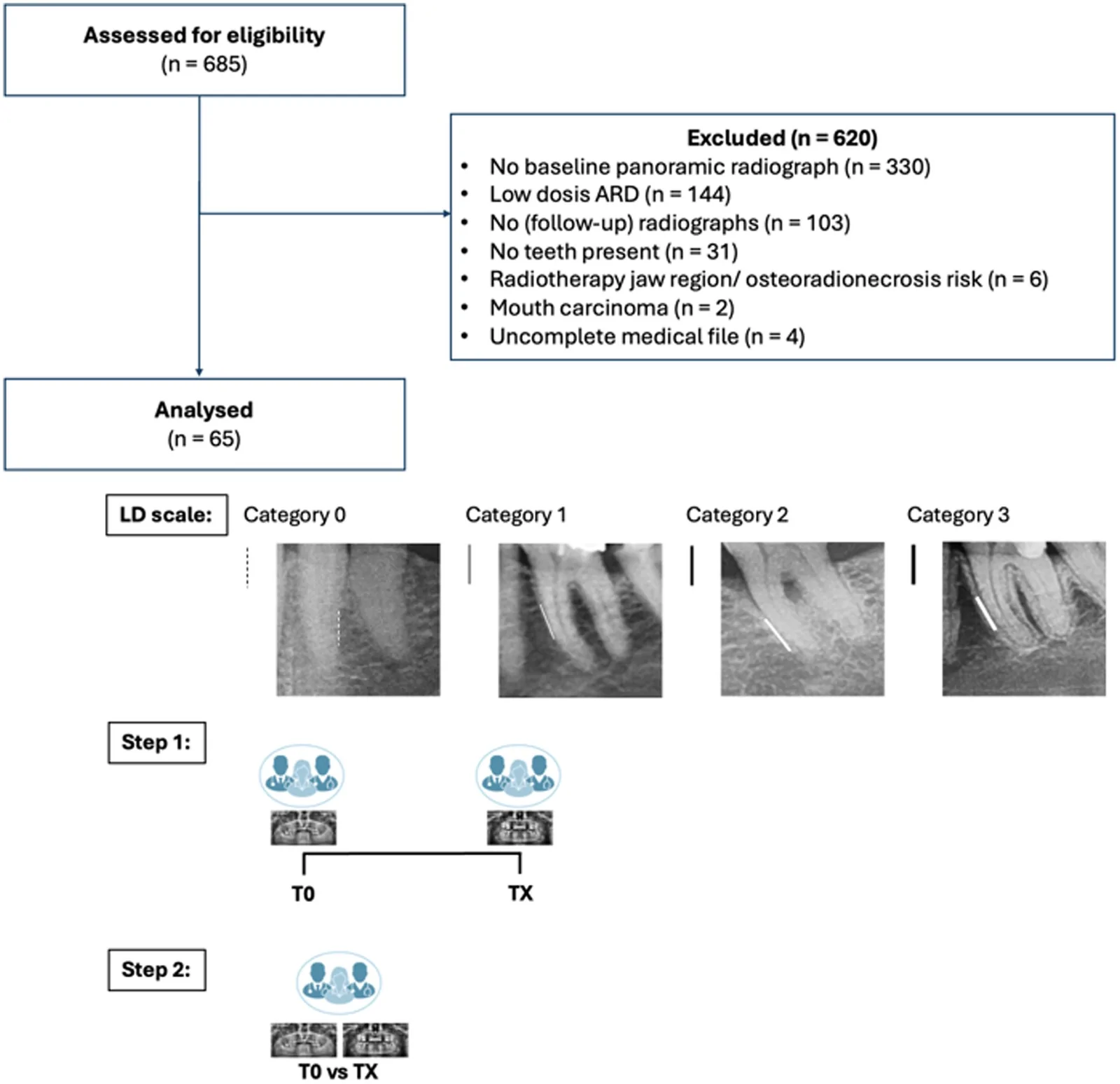
Flow diagram of patient selection for the study cohort. Selected patients were analyzed in 2 steps: (Step 1) panoramic radiographs before ARD use (T0) and after/during ARD use (TX) were randomized, and LD was categorized by 3 blinded oral and maxillofacial radiologists; (Step 2) T0 and TX radiographs were compared side by side to assess LD changes. Abbreviations: ARD = antiresorptive drug; LD = lamina dura.

### Statistical analyses

Statistical analyses were performed using R studio (Posit, Version 2025.05.0 + 496). To calculate the interobserver agreement, Fleiss’ kappa test and weighted kappa were used. The agreement was considered fair when the test result was >0.21-0.40, moderate when >0.41-0.60, substantial when >0.61-0.80, and almost perfect when >0.80.^30^ For the intraobserver agreement, weighted kappa was used.^31^ The between-group differences were analyzed using the Mann-Whitney U test, while within-group changes were assessed using the Wilcoxon signed-rank test. Fisher’s exact test was applied to compare the categorical changes between ARD-treated and control groups. Associations between LD thickening and MRONJ incidence were assessed using a Chi-square test. Kaplan-Meier survival analysis and Spearman correlation analyses were used to explore the relationship between ARD exposure duration and LD thickening as well as the probability of MRONJ development over time stratified by LD status.

## Results

### Participant demographics and clinical features

Of the 685 candidates assessed, 620 were excluded due to missing pre-ARD radiographs, low-dose ARD use, edentulism, maxillofacial radiotherapy, oral carcinoma, or incomplete medical records (Figure 1). A total of 65 eligible cases were included in this study. A control group of 65 individuals was also included. Participant timelines are summarized in Table S1 where among the MRONJ+ patients, the ARD intake was often between 6 and 24 months (40%), followed by 24-36 months (29%), more than 36 months (24%), and less than 6 months (7%). The time from ARD initiation to diagnosis of MRONJ was ≤ 6 months in 24%, 6-24 months in 37%, and more than 24 months for the 39% of cases. In the MRONJ- group, the ARD intake was also often between 6 and 24 months (50%). Longer durations of ARD exposure (> 48 months) were less frequent in all groups. The control group was selected to fit the T0-TX intervals of the ARD-treated group, where also the majority had an T0-TX interval between 6 and 24 months (52%).

Table 1 describes participant characteristics. In the ARD-treated group, breast cancer was the most common primary diagnosis, followed by multiple myeloma. Among the ARD-treated participants, 45 developed MRONJ, while 20 did not. The majority in both MRONJ+ and MRONJ− groups were female (67 and 65%, respectively) compared to male (33 and 35%, respectively). Most patients in both groups were aged between 55 and 75 years. Denosumab was more frequently administered (80% in MRONJ-positive and 65% in MRONJ-negative groups) compared to zoledronic acid (20% and 35%, respectively). In the MRONJ-positive group, 8 were active smokers and 9 had a history of smoking. In contrast, the MRONJ-negative group had no active smokers, and 5 were former smokers. Tooth removal emerged as a notable risk factor: 62% of MRONJ-positive patients had undergone tooth extraction, whereas 65% of MRONJ-negative patients had not (*P* < .05).

**Table 1:** Description of study participants characteristics.

Characteristics		ARD-treated group (*n* = 65)				Control group (*n* = 65)	
		MRONJ+		MRONJ−			
Number, *n*		45	69%	20	31%	N/A	
Sex, *n*	Female	30	67%	13	65%	40	62%
	Male	15	33%	7	35%	25	38%
Age, *n*	40-55	8	18%	2	10%	9	14%
	55-65	12	27%	9	45%	18	28%
	65-75	18	40%	8	40%	16	25%
	75-90	7	15%	1	5%	22	34%
Nicotine usage, *n*	No	27	60%	15	75%	49	75%
	Actively	8	18%	0	N/A	6	9%
	Previously	9	20%	5	25%	10	16%
Alcohol usage, *n*	No	38	85%	18	90%	59	91%
	Yes (> 1 unit/day)	7	15%	2	10%	6	9%
Primary disease, *n*	Multiple myeloma	7	15%	5	25%	N/A	
	Breast cancer	22	49%	11	55%	N/A	
	Prostate cancer	5	11%	1	5%	N/A	
	Lung cancer	3	7%	2	10%	N/A	
	Renal cell cancer	3	7%	0	N/A	N/A	
	Others	5	11%	1	5%	N/A	
Antiresorptive drugs, *n*	Zoledronic acid	9	20%	7	35%	0	
	Denosumab	36	80%	13	65%	0	
MRONJ stage, *n*	Stage 0	8	18%	N/A	N/A	N/A	
	Stage 1	26	58%	N/A	N/A	N/A	
	Stage 2	11	24%	N/A	N/A	N/A	
	Stage 3	0	N/A	N/A	N/A	N/A	
Dental hygiene, *n*	Good	17	38%	5	25%	40	62%
	Average	16	35%	9	45%	20	31%
	Bad	12	27%	6	30%	5	7%
Extractions performed after/during ARD use, *n*	No	17	38%	13	65%	14	22%
	Yes (during/after)	28	62%	7	35%	N/A	
	Yes (no ARD use)	N/A		N/A		51	78%
Polypharmacy	Corticosteroids	11	24%	4	20%	0	
	Chemotherapy	2	5%	1	5%	0	
	Immune therapy	3	7%	3	15%	0	
	Anti-hormonal therapy	21	47%	5	25%	0	
	Targeted therapy (Anti-angiogenic)	15 6	33% 13%	4 2	20% 10%	0	

Abbreviations: ARD = antiresorptive drug; MRONJ = medication-related osteonecrosis of the jaw.

### Effect of ARDs on LD thickness over time

The progressive changes in LD thickness across the different groups (ARD-treated and control) and timepoints (T0, TX, and T0 vs TX) were scored by 3 independent blinded maxillofacial radiologists (Table S2) and a consensus expert agreement score was reached. The overall interobserver agreement for the evaluation of LD thickening was excellent at T0, with a Fleiss’ kappa of 0.828, indicating almost perfect agreement among the 3 radiologists. At TX, the agreement remained high, with a Fleiss’ kappa of 0.736, reflecting substantial agreement. To provide more detailed insight into the interobserver variability, pairwise weighted kappa values were calculated. At T0, agreement between observers was almost perfect across all pairs (observer 1 vs 2: Κ = 0.866; observer 1 vs 3: Κ = 0.900; observer 2 vs 3: Κ = 0.893; all *P* < .001). At T1, agreements remained substantial to almost perfect (observer 1 vs 2: Κ = 0.755; observer 1 vs 3: Κ = 0.859; observer 2 vs 3: Κ = 0.854; all *P* < .001). Light’s pooled weighted kappa supported these findings, with Κ = 0.886 for T0 and Κ = 0.822 for T1. These results suggest that the scoring system used was reliable and reproducible across observers and timepoints. The intraobserver agreement was substantial at both T0 and TX with a weighted kappa of 0.681 and 0.754, respectively.

Between-group differences were tested using the Mann-Whitney U test showing that at T0, no significant difference in LD thickness was observed between the ARD-treated and control groups. However, by TX, a significant difference emerged (*P* < .0001) (Figure 2A). Within-group changes were assessed using Wilcoxon signed rank test indicating that in the ARD-treated group, LD thickness shifted notably; at T0 56.9% of patients had a normal LD, whereas at TX, 55.4% showed mild thickening and 13.8% showed marked thickening (*P* < .0001, r = 0.81). In contrast, the control group remained largely unchanged, with 87.7% showing normal LD at T0 and 89.2% at TX (*P* < .05, r = 0.33) (Figure 2A). Further evaluation by 3 radiologists comparing T0 vs TX radiographs side-by-side revealed that 66% of ARD-treated patients exhibited LD thickening and even 8% severe thickening. In the control group, only 8% showed any thickening, while 92% showed no change (Fisher’s exact test, *P* < .0001) (Figure 2B). Kaplan-Meier analysis was performed to estimate the time to LD thickening in the ARD treated group (Figure S1A). The median time to LD thickening was 27 months, with an estimated survival probability of 0.49 (95% CI, 0.38-0.64). Spearman’s rank correlation between ARD duration and thickening was not significant (*P* = .14). Comparing the ARD-treated group to the control group in a similar analysis, the probability of LD thickening was significantly higher (*P* < .0001) for the ARD-treated group (Figure S1B). Furthermore, a Kaplan-Meier analysis compared the time to LD thickening between patients treated with bisphosphonates (zoledronic acid) and denosumab (Figure S2). The time to LD thickening was shorter in the denosumab treated group and Cox proportional hazard analyses indicated that these patients had a higher risk of LD thickening (HR = 1.91). However, this difference did not reach statistical significance (*P* = .068).

**Figure 2:**
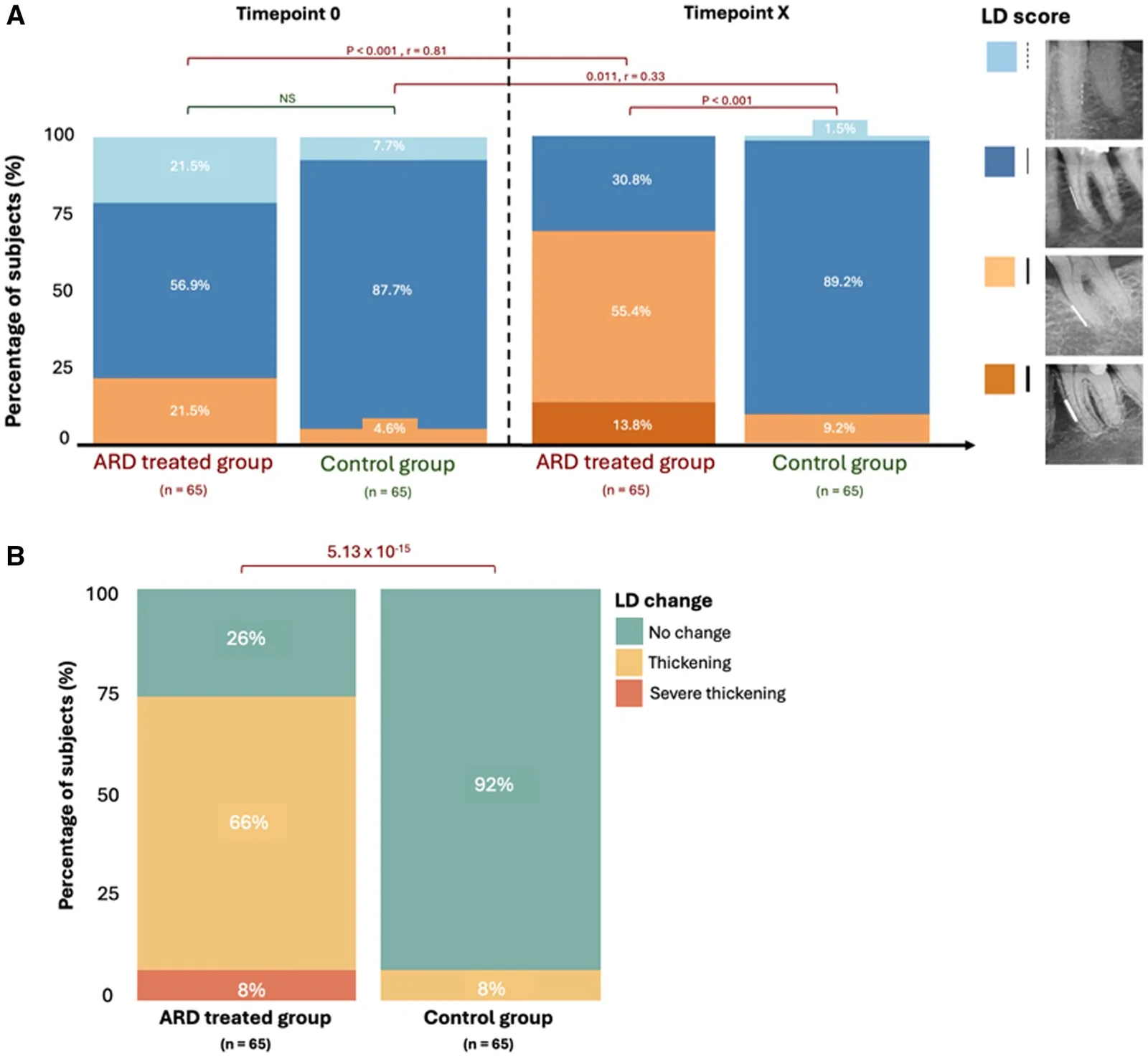
The effect of antiresorptive drugs on the lamina dura. (A) Effect of ARD treatment on lamina dura thickness. Thickness was evaluated at baseline (timepoint 0) and during/after treatment (timepoint X). Between-group differences were tested using Mann-Whitney U test; within-group changes were assessed using the Wilcoxon signed-rank test with effect size (*r*). *P*-values are shown (NS = not significant). (B) Distribution of lamina dura changes over time in ARD treated versus control groups. Lamina dura thickness was evaluated by concurrently reviewing pre- and post-ARD radiographs. Fisher’s exact test indicated a statistically significant difference. Abbreviations: ARD = antiresorptive drug; LD = lamina dura.

The effect of ARD use on LD thickening is illustrated through representative cases (Figure 3). A mild thickening is observed from image A to B, and a marked thickening from C to D. These examples visually demonstrate the variability in LD changes associated with ARD use and MRONJ development. Notably, the first patient (Figure 3, images A-B) exhibited LD thickening without developing MRONJ, while the second patient (Figure 3, images C-D) showed progressive LD thickening and was diagnosed with MRONJ 4 months after the follow-up radiograph. Chi square analyses and Kaplan-Meier analysis (log rank test) of LD thickening on MRONJ development showed no statistically significant association (*P* = .4377, *P* = .39, respectively) (Figure S3).

**Figure 3:**
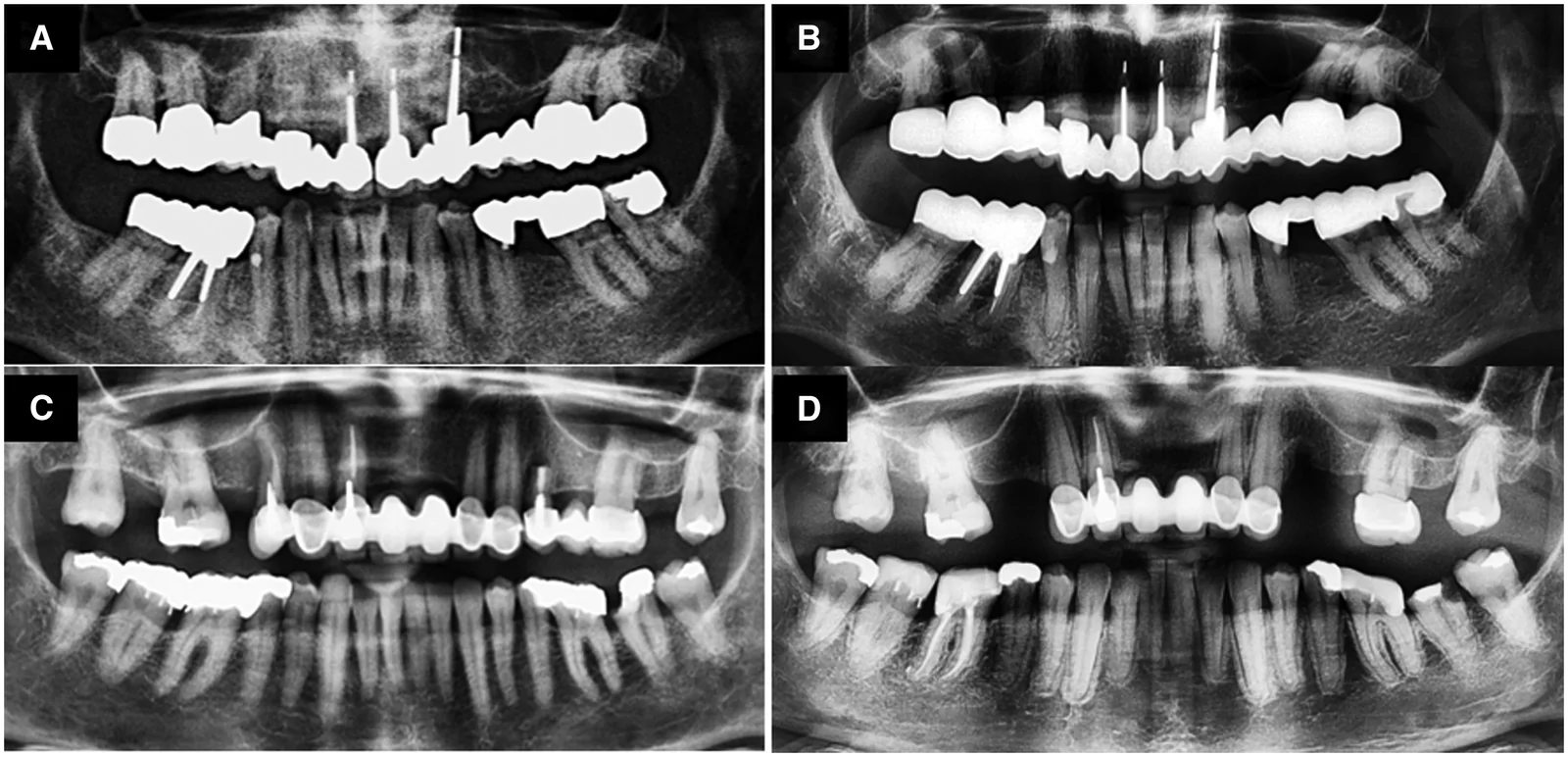
The thickening of the lamina dura in patients using ARD and as a predictive indicator of MRONJ. (A) Panoramic radiograph of patient 1 before ARD use. (B) Panoramic radiograph of patient 1 taken after using ARD for 24 months showing mild thickening (category 2). (C) Panoramic radiograph of patient 2 before ARD use. (D) Panoramic radiograph of patient 2 taken 4 months before developing MRONJ stage 1 after using ARD for 47 months showing marked thickening (category 3).

## Discussion

The use of ARDs, including traditional bisphosphonates and denosumab, is expected to rise in response to the growing prevalence of oncological conditions and bone-related disorders such as osteoporosis and metastatic bone disease.^27^^,^^28^ While ARDs are effective in managing bone-related conditions, they can lead to MRONJ, impacting patients’ quality of life both during treatment and in survivorship.^32^ Identifying and validating radiological risk factors is a key step in preventing MRONJ and improving the quality of life of these patients during, but also after ARD use. The primary aim of this study was to investigate the hypothesis that high-dose intravenous ARD treatment contributes to the thickening of the LD, thereby supporting its potential use as a predictor for MRONJ risk. A subobjective was to examine the association between LD thickening and MRONJ incidence. For this purpose, the present retrospective longitudinal cohort was based on a case-control design, assessing panoramic radiographs patients’ before and after ARD use as compared to panoramic radiographs acquired for dental treatment in age- and sex-matched control subjects without any use of medication over time.

The LD appears on radiographic images as the cortical bone lining the tooth root, connecting the PDL space to the alveolar bone. It is often one of the first structures affected by changes in bone metabolism as seen in systematic conditions such as Paget’s disease.^33^^,^^34^ We hypothesized that ARDs could cause LD thickening, since ARDs affect the bone metabolism by inhibiting osteoclast activity.^35^^,^^36^ This results in a denser bone structure and thickening of the LD which could impair local blood flow, thereby limiting the delivery of essential nutrients and immune cells. This compromised vascular environment may negatively affect soft tissue function, healing capacity, and immune response—all critical in preventing osteonecrosis (Figure 4). The data of this study provide evidence that the use of ARDs is strongly associated with radiological LD thickening over time. While LD thickening has previously been noted during MRONJ diagnosis, this study is, to our knowledge, the first to assess LD changes on baseline panoramic radiographs prior to ARD use.

**Figure 4:**
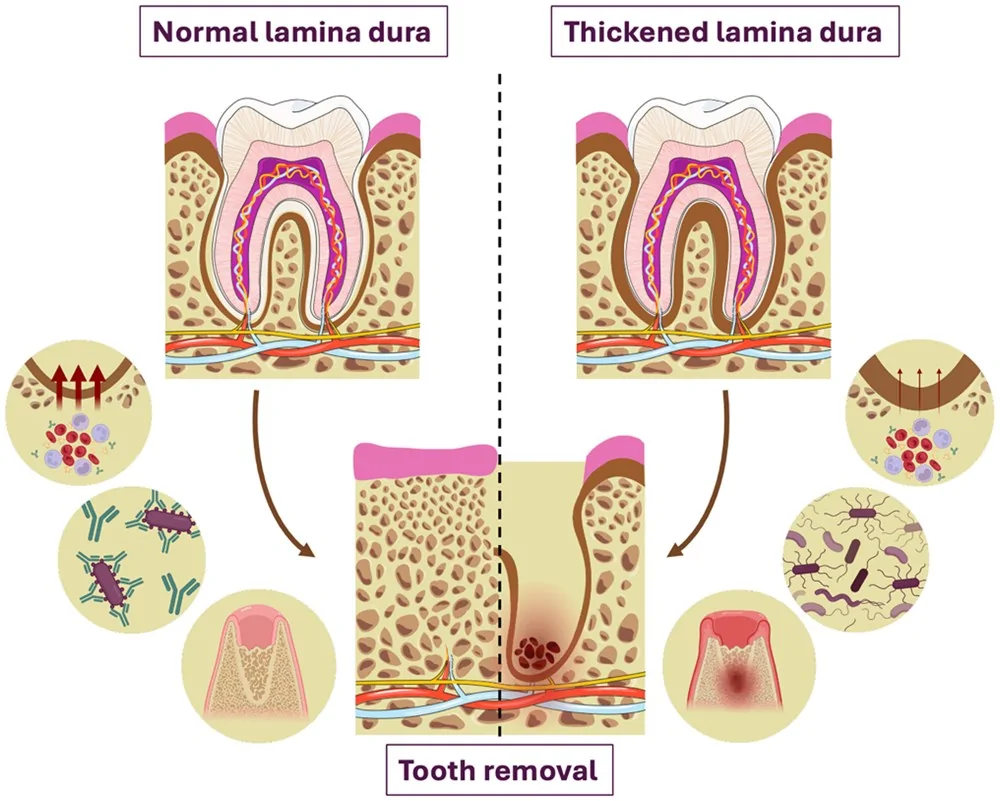
Proposed role of LD thickening in the pathogenesis of MRONJ. Lamina dura thickening may reduce vascularity and impair the delivery of nutrients and immune cells, negatively affecting tissue function, healing capacity, and immune response. When combined with tooth removals, this compromised environment may further increase the risk of MRONJ development.

Investigating this association may reveal whether LD thickening could serve as an early indicator of increased MRONJ risk. This study did not demonstrate a significant association between LD thickening and MRONJ, similar to several previous investigations that compared MRONJ-positive and MRONJ-negative ARD users.^2^^,^^37^^,^^38^ Previous studies of this sort often compared MRONJ+ patients with MRONJ− ARD users or with healthy control subjects.^2^^,^^23^^,^^37^ This limits the ability to determine whether radiographic alterations, such as LD thickening, were present before ARD exposure as this sort of study design does not establish baseline conditions. The findings of this study suggest that LD changes are primarily a consequence of ARD exposure and may represent a risk indicator rather than a definitive predictive marker for MRONJ. Medication-related osteonecrosis of the jaw development is multifactorial and can be influenced by additional factors such as genetic predisposition and local triggers.^39–42^ Tooth extraction remains a well-established primary risk factor, and the combination of LD thickening with extraction could increase MRONJ risk.^43^ Therefore, LD changes should be considered in clinical decision-making. This study was not able to show a significant association between LD status and the probability of MRONJ. However, it is important to note that not all patients in the MRONJ-negative group had undergone a tooth extraction, and still carried a residual risk of MRONJ following ARD therapy. Furthermore, our study revealed that the probability of LD thickening increased faster in the denosumab-treated group than in the bisphosphonate-treated group. Although this difference did not reach statistical significance, it aligns with the existing evidence that denosumab is more potent and more frequently associated with MRONJ.^44^^,^^45^

Currently, protocols for dental extractions in patients receiving ARDs vary across clinical practices and are not universally standardized. The thickening of the LD is a risk indicator that can be easily detected on routine panoramic radiographs and assist clinicians in the decision-making process and aid the referral strategy should tooth removal be needed. Recognizing this feature may prompt preventive measures in the protocols such as preoperative antibiotic therapy and the use of LPRF, potentially lowering the risk of MRONJ in patients presenting this radiographic risk indicator, thereby contributing to improved patient wellbeing.^46^^,^^47^ Furthermore, such knowledge can help in the decision-making of inducing a drug holiday or lowering the ARD dose in case such LD thickening is observed. Lastly, this paves the way for exploring artificial intelligence (AI)-driven identification of LD thickening as a supportive tool for clinicians when referring and treating ARD patients who require tooth extractions. While clinicians are trained to recognize such changes, the assessment of LD thickening can be partly subjective and may show inter-observer variability. An AI-based tool could therefore help reduce variability and ensure subtle findings are not overlooked. Furthermore, in clinical practice, it remains important to avoid patient positioning errors, especially in longitudinal comparisons, as these can influence the radiographs, particularly anteriorly.^48^

Limitations to this study include its retrospective design, which introduced variability in the timelines between baseline and follow-up radiographs. Additionally, as a single-center study, the sample size was relatively limited. Lamina dura thickening was assessed using panoramic radiographs, which are prone to superimposition, while CBCT eliminates such overlap. However, this approach reflects real-world clinical practice, in which panoramic imaging is the primary imaging modality, typically performed opportunistically during dental extraction visits, rather than at standardized intervals or using CBCT. Before LD thickening can be considered a clinical predictive marker, further validation through larger, multicenter, prospective studies focusing on long-term ARD exposure is needed.

## Conclusions

The findings of this clinical retrospective longitudinal cohort study present that the use of high-dose ARDs is strongly associated with progressive radiographic LD thickening. This supports the hypothesis that LD thickening reflects ARD-related bone remodeling and may serve as a radiological risk indicator for MRONJ susceptibility. In this cohort, LD thickening was not directly associated with MRONJ development and its role as an early marker could not be confirmed. Given the clear association with ARD exposure, LD thickening remains a feature of interest that could contribute to risk stratification when combined with other clinical factors. Future studies with larger cohorts and longitudinal designs are needed to clarify its potential value.

## Supplementary Material

twag029_Supplementary_Data
