# Supplementary material for: Lamina dura thickening in patients on antiresorptive therapy as a radiographic risk indicator of medication-related osteonecrosis of the jaw: a retrospective longitudinal cohort study
Source: Dentomaxillofac Radiol. 2026 May 19;55(7):722–31. doi: 10.1093/dmfr/twag029 (PMC13624511; doi:10.1093/dmfr/twag029)
Supplement: Supplementary file 1 [file twag029_supplementary_data.docx]

**Supplementary figures and tables:**

**Supplementary table 1.** Timeline description of the participants

| **Timeline** | | **ARD-treated group (n = 65)** | | | | | **Control group (n = 65)** |
| --- | --- | --- | --- | --- | --- | --- | --- |
|  | | **MRONJ +** | | **MRONJ -** | | |  |
| **Number, n** |  | 45 |  | 20 |  | | 65 |
| **Time ARDs intake (months), n** | ≤ 6 | 3 | 7% | 2 | | 10% | N/A |
|  | >6 - ≤24 | 18 | 40% | 10 | | 50% | N/A |
|  | >24 - ≤36 | 13 | 29% | 3 | | 15% | N/A |
|  | >36 | 11 | 24% | 5 | | 25% | N/A |
| **Time ARD start – MRONJ (months), n** | ≤ 6 | 11 | 24% | N/A | |  | N/A |
|  | >6 - ≤24 | 17 | 37% | N/A | |  | N/A |
|  | >24 - ≤36 | 6 | 13% | N/A | |  | N/A |
|  | >36 | 11 | 26% | N/A | |  | N/A |
| **Time T0 – TX (months), n** | ≤ 6 | 2 | 4% | 1 | | 5% | 11 17% |
|  | >6 - ≤24 | 18 | 40% | 9 | | 45% | 34 52% |
|  | >24 - ≤36 | 9 | 20% | 5 | | 25% | 4 6% |
|  | >36 | 16 | 36% | 5 | | 25% | 16 25% |

**Supplementary table 2.** The description of the Lamina Dura over time.

| **Characteristics** | | **ARD-treated group (n = 65)** | | | | | **Control group (n = 65)** |
| --- | --- | --- | --- | --- | --- | --- | --- |
|  | | **MRONJ +** | | **MRONJ -** | |  | |
| **Number, n** |  | 45 |  | 20 |  | 65 | |
| **Lamina dura assessment at T0** | Category 0   | 10 22% | | 4 20% | | 5 8% | |
|  | Category 1   | 26 58% | | 13 65% | | 57 88% | |
|  | Category 2   | 9 20% | | 5 25% | | 3 4% | |
|  | Category 3   | 0 N/A | | 0 N/A | | 0 N/A | |
| **Lamina dura assessment at TX** | Category 0   | 0 N/A | | 0 N/A | | 1 2% | |
|  | Category 1   | 14 31% | | 6 30% | | 58 89% | |
|  | Category 2   | 25 56% | | 11 55% | | 6 9% | |
|  | Category 3   | 6 13% | | 3 15% | | 0 N/A | |
| **Lamina dura assessment T0 vs TX** | No change  Thickening  Severe thickening | 10 22%  32 71%  3 7% | | 7 35%  11 55%  2 10% | | 60 92%  5 8%  0 N/A | |

**A**

**B**

***Supplementary figure 1. Kaplan-Meier curve showing the probability of lamina dura thickening over time in patients using antiresorptive drugs and control subjects. (A)*** *The median time to LD thickening in the patient group was 27 months, with an estimated survival probability of 0.49 (95% CI: 0.38–0.64). Spearman correlation between ARD duration and thickening was non-significant (P > 0.05). The x-axis represents ARD use duration (in months), and the y-axis represents probability of lamina dura thickening (%).* ***(B)*** *The case group (ARD-treated group) show a higher incidence of thickening throughout follow-up, with a significant difference compared to the control group (P < 0.0001).*

***Supplementary figure 2. Kaplan-Meier curve showing the probability of lamina dura thickening over time in patients using two types of antiresorptive drugs.*** *Zoledronic acid (bisphosphonates, blue) and denosumab (orange). The x-axis represents ARD use duration (in months), and the y-axis represents the probability of lamina dura thickening (%). The table below indicates the number of patients at risk at each time point based on medication type. Cox proportional hazards analysis was performed (HR = 1.91, p = 0.068).*

**A**

**B**

***Supplementary figure 3. Correlation between LD-thickening and MRONJ incidence. (A)*** *Chi square analysis of LD thickening on MRONJ incidence. (P > 0.05).* ***(B)*** *Kaplan-Meier curve illustrating the probability of MRONJ development over time in patients using ARDs (in months) stratified by LD status (no change, thickening). The Y-axis shows the cumulative probability of MRONJ (%). (P = 0.39)*
